# Association between exercise habits in adolescence and old age and the risk of mild cognitive impairment: the Bunkyo health study

**DOI:** 10.3389/fnagi.2024.1456665

**Published:** 2024-11-11

**Authors:** Huicong Shi, Hiroki Tabata, Hikaru Otsuka, Takahito Iwashimizu, Hideyoshi Kaga, Yuki Someya, Abulaiti Abudurezake, Saori Kakehi, Hitoshi Naito, Yasuyo Yoshizawa, Ryuzo Kawamori, Hirotaka Watada, Yoshifumi Tamura

**Affiliations:** ^1^Department of Sports Medicine and Sportology, Juntendo University, Graduate School of Medicine, Bunkyo ku, Tokyo, Japan; ^2^Sportology Center, Juntendo University, Graduate School of Medicine, Bunkyo Ku, Tokyo, Japan; ^3^Juntendo Advanced Research Institute for Health Science, Bunkyo ku, Tokyo, Japan; ^4^Juntendo University Urayasu Hospital, Urayasu city, Chiba, Japan; ^5^Department of Metabolism & Endocrinology, Juntendo University, Graduate School of Medicine, Bunkyo ku, Tokyo, Japan; ^6^Juntendo University Graduate School of Health and Sports Science, Inzai-shi, Chiba, Japan; ^7^Faculty of International Liberal Arts, Juntendo University, Bunkyo ku, Tokyo, Japan

**Keywords:** physical activity, cognitive function, regional brain volumes, blood markers, cross-sectional study

## Abstract

**Background:**

Exercise in adolescence and old age improves cognitive function in older adults, but the combined effect of exercise habits in both periods is controversial. This study aimed to clarify the relationship between exercise habits in adolescence and old age and mild cognitive impairment (MCI) and to compare regional brain volumes and blood biochemical markers associated with cognitive function in older adults.

**Methods:**

Baseline data of 1615 participants aged 65–84 years from the Bunkyo Health Study were analyzed. MCI was diagnosed using the Japanese version of the Montreal Cognitive Assessment. Participants were divided into four groups based on their exercise habits in adolescence (13–18 years) and old age: no exercise in either period (None-None), exercise in adolescence only (Active-None), exercise in old age only (None-Active), and exercise in both periods (Active-Active). Logistic regression models estimated the odds ratios (ORs) of MCI prevalence. Regional brain volumes, such as the prefrontal cortex, temporal lobe, parietal cortex, and hippocampus, and blood biochemical markers, such as BDNF, IGF-1, and homocysteine, were compared between groups.

**Results:**

The OR for MCI was significantly lower in the Active-Active group than in the None-None group (OR, 0.62; 95% CI, 0.41–0.94). However, there were no significant differences in regional brain volumes and blood biochemical markers between the Active-Active and None-None groups.

**Conclusions:**

Older adults with exercise habits in both adolescence and old age have a lower risk of MCI. However, specific regional brain volumes and biochemical markers may not be associated with differences in cognitive function in older adults.

## Introduction

Long-term care (LTC) is becoming increasingly significant in developed countries with the increasing aging of their populations (Alzheimer’s Association, 2019). Among the various conditions that require LTC, dementia stands out because of its increasing prevalence and impact around the world. Approximately 135 million people worldwide are expected to develop dementia by 2050 (Prince et al., 2020). Recent studies have highlighted mild cognitive impairment (MCI) as a critical precursor to dementia (Bruscoli and Lovestone, 2004; Petersen, 2004; Vos et al., 2015). People with MCI have a higher risk of developing dementia than those without MCI. Specifically, approximately 10–15% of people diagnosed with MCI will develop dementia (Farias et al., 2009). These statistics suggest that strategies aimed at preventing or delaying MCI could significantly reduce the incidence of dementia and consequently, lessen the burden on LTC systems. This preventive approach is crucial in addressing the challenges posed by the increasing incidence of dementia in aging societies.

The WHO guideline recommends physical activity, including exercise, for adults aged 18 and older to help maintain cognitive function and reduce the risk of cognitive decline. Specifically, for older adults over 65 years, the guideline recommends engaging in at least 150 min of moderate-intensity or 75 min of vigorous-intensity aerobic exercise per week, along with resistance training on two or more days per week (World Health Organization, 2019). Indeed, having exercise habits later in life can reduce the risk of developing incident MCI in old age (Forbes et al., 2019; Krell-Roesch et al., 2021). An observational study further found that engaging in vigorous physical activity more than three days per week or moderate physical activity more than five days per week can lower the risk of progressing from MCI to dementia (Kim et al., 2020).

It has been reported that engaging in exercise during early adulthood could enhance cognitive reserve (Greene et al., 2019), which equips the brain with greater flexibility to manage aging and pathological changes (Stern et al., 2020). Studies have shown that higher levels of aerobic fitness in adolescence are associated with increased volumes of key brain regions, such as the hippocampus and prefrontal cortex, which are involved in memory and executive function (Herting et al., 2016; Herting and Nagel, 2012), as well as positive effects on the white matter microstructure, particularly in pathways like the corticospinal tract, which are important for motor behavior (Herting et al., 2014). These findings suggest that aerobic exercise may support brain development during adolescence (Herting and Chu, 2017), potentially enhancing cognitive reserve and contributing to resilience against aging-related cognitive decline. In addition to these findings, a previous prospective study also showed that regular exercise at low (less than 1–2 h/week) or moderate frequency (3–9 h/week) during early life (15–25 years) can delay cognitive function decline in later years (ages 65 and above) (Dik et al., 2003). Dik et al. suggested that this delay may be related to potential mechanisms such as enhanced brain reserve capacity and the stimulation of neurotrophic factors. These findings indicate that having exercise habits in both adolescence and old age may contribute to the prevention of cognitive decline in later life.

Conflicting findings exist regarding the relationship between exercise habits in adolescence and old age and the risk of cognitive decline. A previous study found that individuals who engaged in high levels of leisure-time physical activity (LTPA), such as vigorous activities like aerobic dancing, jogging, and playing handball, or moderate activities like bicycling, swimming, and tennis, in both adolescence and old age had a significantly lower prevalence of Alzheimer’s disease compared to those with low levels of physical activity in either period (Ogino et al., 2019). However, another study showed that older women who were inactive in their teens but became active in their 50s or later had a lower odds ratio for MCI compared with those who were inactive throughout their lives, while those who were active both in their teens and later in life showed no significant difference (Middleton et al., 2010). These conflicting findings highlight the need for further research to clarify the relationship between exercise habits in adolescence and old age and the risk of MCI.

High levels of exercise, particularly aerobic exercise, are correlated with increased brain volume in regions crucial for cognitive function, such as the hippocampus (Firth et al., 2018), prefrontal cortex (Basso et al., 2015), and temporal lobe (Bugg and Head, 2011). For example, a study by Erickson et al. demonstrated that one year of aerobic exercise, specifically 40 min of walking, three times per week, led to approximately a 2% increase in hippocampal volume compared to a control group who did not engage in exercise (Erickson et al., 2011). These anatomical enhancements are believed to be the underlying mechanism for the improved cognitive performances observed in older adults who maintain active lifestyles (Colcombe et al., 2006). Additionally, previous reports have shown that exercise influences key biochemical markers linked to cognitive function, such as brain-derived neurotrophic factor (BDNF) (Piepmeier and Etnier, 2015), insulin-like growth factor 1 (IGF-1) (Tsai et al., 2015), and homocysteine(Setién-Suero et al., 2016). Specifically, elevated levels of BDNF and IGF-1, both associated with neural growth and health, have been observed following regular exercise. Elevated BDNF (Cefis et al., 2023) and IGF-1 (Stein et al., 2021)levels may mitigate the neurodegenerative processes that lead to MCI and dementia.

Therefore, we conducted this study to clarify the relationship between exercise habits in adolescence and old age and the prevalence of MCI among community-dwelling older adults. Furthermore, we examined changes in the volumes of brain regions critical for cognitive function and measured key biochemical markers, such as BDNF, IGF-1, and homocysteine, which are implicated in neurodegenerative processes. This comprehensive approach will expand our understanding of how lifelong exercise influences neurochemical pathways and brain structure, thereby affecting the risk of cognitive decline. Our findings may provide clearer insights into the mechanisms by which exercise benefits cognitive health and help inform the development of more effective prevention strategies for MCI and dementia.

## Materials and methods

### Study design and participants

This cross-sectional study was conducted using baseline data from the Bunkyo Health Study, which was originally designed to investigate the association between skeletal muscle function and the risk factors, including cognitive decline, of needing for LTC in older adults (Someya et al., 2019). A total of 1,629 older adults aged 65–84 years living in Bunkyo-Ku, an urban area in Tokyo, were enrolled in the Bunkyo Health Study. While the original sample size was calculated to evaluate associations such as cognitive decline and muscle mass, the current study is a sub-analysis focusing on different variables, and the sample size for this analysis was not determined based on the original calculation. All the participants completed two-day examinations at the Sportology Center between November 16, 2015, and October 1, 2018. The participants underwent comprehensive assessments, including the Japanese version of the Montreal Cognitive Assessment (MoCA-J) test, brain lesion evaluations performed using magnetic resonance imaging (MRI), physical fitness tests, body composition analysis performed using dual-energy X-ray absorptiometry, and blood biochemical marker analysis. The study protocol was approved by the Ethics Committee of Juntendo University in September 2015 (first approval no. 2015061 and the latest revised version no. M15-0057-M08). The study was conducted in accordance with the principles of the Declaration of Helsinki. All participants provided written informed consent and were informed that they had the right to withdraw from the trial at any time.

**FIGURE 1:**
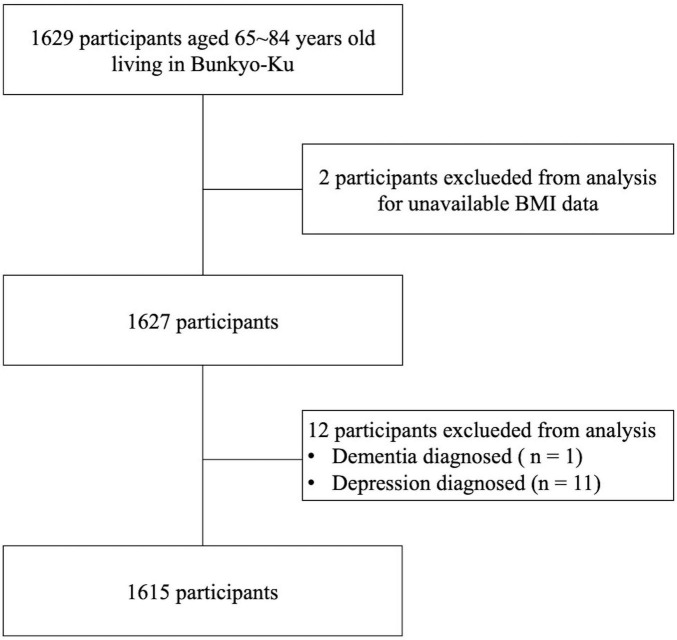
Flow chart of this study population.

Among the 1629 participants enrolled in the Bunkyo Health Study, two participants with unavailable BMI data were excluded. In addition, one was excluded from the present study due to a self-reported diagnosis of dementia. Furthermore, 11 participants who were diagnosed with depression were excluded because depression is an important causative factor of dementia. Finally, 1,615 participants (684 men and 931 women) were included in the analysis (Figure 1).

### Measurements

#### Diagnosis of MCI

Cognitive function and the prevalence of MCI were measured using the MoCA-J test. The MoCA-J has been reported to have a high sensitivity and specificity for detecting MCI (Fujiwara et al., 2010). The MoCA-J includes visuospatial/executive, naming, attention, language, abstraction, delayed recall, and orientation domains, which were scored on a scale of 30 points. If a participant has an education level of 12 years or less, one point is added after the examination (Nasreddine et al., 2005). Notably, recent re-evaluations have indicated that this definition of MCI is more reliable than another screening tool (Ciesielska et al., 2016; Tsoi et al., 2017). Participants with a MoCA-J score of ≤ 22 points were defined as having MCI. Although the commonly used cut-off score is 26/27, previous research suggests that using a lower cut-off, such as 22/23, may provide improved sensitivity and specificity for detecting MCI (Carson et al., 2018; Nara et al., 2018). This approach also aligns with the protocol of the Bunkyo Health Study (Someya et al., 2019), and was therefore selected for use in our study.

#### Definition and classification of exercise habits

Data on exercise habits were self-reported in the baseline questionnaire survey of the Bunkyo Health Study. The participants were divided into four groups based on their exercise habits in adolescence or old age. Those who answered “yes” to the question “Did you participate in Bukatsudo during junior school or senior school?” were defined as those who had exercise habits in adolescence. A traditional sports club in Japanese junior high and high schools called “Bukatsudo” is an important aspect of education in Japanese schools (Cave, 2004). Although participation in Bukatsudo is not compulsory, it is incorporated into the educational framework. Those who joined Bukatsudo practiced at least six times per week for at least 3–3.5 h/day in school even while on vacation (Gero et al., 2018). Those who responded “yes” to the question “Do you currently have exercise habits?” were described as those who exercise in old age.

It should be noted that participation in structured sports club activities such as Bukatsudo in adolescence involved regular, high-frequency exercise, which differ from the exercise habits of older adults, who engage in various types of exerises with different frequencies and intensities. Specific types of sports in adolescence and old age are shown in Supplementary Tables 1, 2, respectively. The participants were categorized into the following four groups based on their responses: None-None group (no exercise habits in adolescence nor old age), None-Active group (no exercise habits in adolescence, but exercises in old age), Active-None group (exercise habits in adolescence, but not in old age), and Active-Active group (exercise habits in both adolescence and old age).

#### Evaluation of the brain volume using MRI

All participants underwent whole-brain MR performed using a 0.3 T clinical MR scanner (AIRIS Vento, Hitachi, Tokyo, Japan). Primary brain MRI evaluation was assessed by an experienced neuroradiologist based on axial T2-weighted images and FLAIR images obtained using the same 0.3 T clinical MR scanner. Additionally, 0.3 T 3D T1-weighted images were used for the quantification of both whole and regional brain volumes (Murata et al., 2022). We collated data on intracranial volume (ICV), including the volumes of specific regions associated with cognitive function, such as the prefrontal cortex, temporal lobe, parietal cortex, and hippocampus. However, seven participants with missing data on brain volumes were excluded from the analysis of regional brain volumes. We corrected the measured volumes of each brain region using the ICV to control for variations in ICV among the participants. All statistical analyses were conducted using data expressed as percentages of the ICV.

#### Other measurements

Height was measured with the participant in an upright position. The measurement was made within 0.1 cm using a stadiometer (YS-201-P; YAGAMI Inc., Nagoya, Japan). Body mass was measured within 0.1 kg using an electronic scale (InBody770; Biospace, Seoul, Korea). Self-administered questionnaires were used to collect information on age (years), years of education (years), and smoking status (current and past). Dietary intake was assessed using a Brief self-administered Diet History Questionnaire to measure alcohol intake (Kobayashi et al., 2012). Physical activity levels were evaluated using the International Physical Activity Questionnaire (IPAQ) (Craig et al., 2003; Murase, 2003). We used the short version of the Geriatric Depression Scale to assess depression or depression tendency, which was defined as a score of ≥ 5 points. Medical history and medication information were recorded by a physician in the interviews using a semi-structured questionnaire, which included conditions such as cerebrovascular disease and stroke. After an overnight fast, blood samples for relevant biochemical tests were collected in the morning. Blood glucose and hemoglobin A1C levels were measured at the Commissioned Clinical Laboratory Center (SRL Inc., Tokyo, Japan). Diabetes mellitus was defined as a fasting blood glucose level ≥ 126 mg/dL and/or a two-h blood glucose level ≥ 200 mg/dL after a 75-g oral glucose tolerance test, and a hemoglobin A1C level ≥ 6.5%, or current use of diabetes medication. Dyslipidemia was defined as low-density lipoprotein (LDL) cholesterol ≥ 140 mg/dL, high-density lipoprotein (HDL) cholesterol < 40 mg/dL, triglycerides ≥ 150 mg/dL, or the current use of lipid-lowering agents. Hypertension was defined as taking antihypertensive drugs or having a systolic blood pressure 140 mmHg and a diastolic blood pressure 90 mmHg. Serum BDNF level was measured using a multiplex assay (MILLIPLEX MAP Human Myokine Magnetic Bead Panel; Merck, Darmstadt, Germany). IGF-1 and homocysteine concentrations were measured using radioimmunoassay and high-performance liquid chromatography, respectively.

#### Statistical analysis

Data are presented as medians (quartiles) for continuous variables and percentages for categorical variables. Logistic regression models were used to estimate the odds ratio (ORs) and 95% confidence interval (CIs) for the prevalence of MCI in each group compared with the None-None group. Model 1 was adjusted for age (continuous variable) and sex (men or women). Model 2 was adjusted for the age and sex plus body mass index (BMI) (continuous variable) and years of education (continuous variable). Model 3 was adjusted for the variables adjusted in Model 2 plus current and past smoking status (yes or no), hypertension (yes or no), diabetes mellitus (yes or no), cerebrovascular disease (yes or no), and alcohol intake (continuous variable).

Differences in regional brain volumes and BDNF, IGF-1, and homocysteine concentrations among the four groups were compared using analysis of covariance adjusted for the following potential confounders: age, sex, BMI, years of education, smoking status (current and past), hypertension, diabetes mellitus, cerebrovascular disease, and alcohol intake. We adjusted for multiple comparisons using post-hoc Bonferroni correction. Values are presented as means ± standard error.

IBM SPSS Statistics for Windows, version 29.0. (IBM Corp., Armonk, NY, USA) was used for all analyses. All statistical tests were two-sided and conducted at a significance level of 5%.

A post-hoc power analysis was performed using the G*Power 3.1 to evaluate whether our data had sufficient verification power.

## Results

### Demographic and baseline characteristics of the participants

The eligible participants included 685 men and 930 women. Table 1 shows the detailed demographic and baseline characteristics of the participants classified into four groups according to their exercise habits in adolescence and old age. The None-None and None-Active groups had relatively high and similar numbers of women. The Active-Active group had a relatively low prevalence of hypertension and MCI.

**TABLE 1:** Characteristics of the participants and comparison of the four groups according to exercise habits in adolescence and old age.

	Overall (*N* = 1615)	None-None (*N* = 249)	None-Active (*N* = 499)	Active-None (*N* = 302)	Active-Active (*N* = 565)
Age, years	73 (68–77)	73 (69–77)	74 (69–78)	72 (68–77)	72 (68–77)
Numbers of women, *n* (%)	931 (57.6)	162 (65.1)	333 (66.7)	148 (49.0)	288 (51.0)
Years of education, years	14 (12–16)	14 (12–16)	14 (12–16)	14 (12–16)	14 (12–16)
Past smoking status, *n* (%)	663 (41.1)	81 (32.5)	162 (32.5)	149 (49.3)	271 (48.0)
Current smoking status, *n* (%)	122 (7.6)	17 (6.8)	21 (4.2)	34 (11.3)	50 (8.8)
Alcohol intake, g/d	1.1 (0.0–16.7)	0.3 (0.0–14.7)	0.5 (0.0–10.3)	1.4 (0.0–23.9)	3.5 (0.0–23.5)
Physical activity level (METs hour/week)	29.9 (16.5–54.2)	23.1 (11.6–40.0)	34.1 (19.8–56.9)	20.4 (11.0–38.6)	35.4 (35.4–61.4)
Height, cm	157.2 (151.7–164.7)	155.7 (151.0–162.3)	154.5 (150.5–162.0)	160.1 (153.0–166.4)	159.2 (152.6–166.0)
Weight, kg	57.2 (50.4–65.2)	57.7 (51.0–65.6)	54.7 (48.8–62.3)	61.3 (52.3–68.3)	58.0 (51.1–65.8)
Body mass index, kg/m^2^	23.0 (21.0–25.1)	23.6 (21.7–25.6)	22.6 (20.7–24.6)	23.4 (21.6–25.6)	22.9 (21.0–25.0
MoCA-J score	26 (23–27)	26 (23–28)	26 (23–27)	25 (23–27)	26 (24–27)
MCI, *n* (%)	292 (18.1)	57 (22.9)	91 (18.2)	60 (19.9)	84 (14.9)
Hypertension^a^, *n* (%)	1067 (66.1)	178 (71.5)	322 (64.5)	212 (70.2)	355 (62.8)
Diabetes mellitus^b^, *n* (%)	208 (12.9)	30 (12.0)	55 (11.0)	51 (16.9)	72 (12.7)
Cerebrovascular disease ^c^, *n* (%)	67 (4.1)	17 (6.8)	20 (4.0)	9 (3.0)	21 (3.7)
Dyslipidemia^d^, *n* (%)	1013 (62.7)	165 (66.3)	305 (61.1)	199 (65.9)	344 (60.9)
Geriatric depression^e^, *n* (%)	333 (20.6)	82 (32.9)	96 (19.2)	71 (23.5)	84 (14.9)
Stroke^f^, *n* (%)	40 (2.5)	10 (4.0)	11 (2.2)	7 (2.3)	12 (2.1)

METs, Metabolic Equivalent of Task from the Framingham Physical Activity Index; MoCA-J, the Montreal Cognitive Assessment, Japanese version. MCI, mild cognitive impairment. Results are presented as the median (quartile) or n (%). ^a^Hypertension was defined as taking antihypertensive drugs or having a systolic blood pressure ≥ 140 mmHg and a diastolic blood pressure ≥ 90 mmHg. ^b^Diabetes was defined as taking hypoglycemic drugs or meeting the diabetes criteria according to the Japan Diabetes Society 2010 classification (hemoglobin A1c ≥ 6.5 and fasting blood glucose ≧126 mg/dl, and/or a 2-h glucose level ≥ 200 mg/dl after the 75-g oral glucose tolerance test). ^c^Cerebrovascular disease was defined as a self-reported diagnosis until baseline measurements were taken. ^d^Dyslipidemia was defined as low-density lipoprotein (LDL) cholesterol ≥ 140 mg/dL, high-density lipoprotein (HDL) cholesterol < 40 mg/dL, triglycerides ≥ 150 mg/dL, or the current use of lipid-lowering agents. ^e^Geriatric depression was assessed by The short version of the Geriatric Depression Scale (GDS-15), and five or higher GDS-15 scores are defined as geriatric depression. ^f^Stroke was recorded as part of the medical history through a questionnaire.

### Association between exercise habits and the prevalence of MCI

The associations between exercise habits in adolescence and old age and the prevalence of MCI are shown in Table 2. The Active-Active group had the lowest proportion of MCI (14.9%). Using the None-None group as a reference, the odds ratio for the Active-Active group was significantly lower MCI risk after adjusting all confounding factors (OR, 0.62; 95% Cl, 0.41–0.94). Additionally, The ORs for MCI in the None-Active and Active-None groups were not significant; however, both groups had a slightly lower risk of MCI than the None-None group after adjustment of all confounding factors. A post-hoc power analysis revealed that the sample size had a power over 95%. However, post-hoc power analysis indicated a power of 50% in the None-None group, 75% in the None-Active group, 56% in the Active-None group and 80% in the Active-Active group, respectively.

**TABLE 2:** Associations between exercise habits in adolescence and old age and the prevalence of MCI.

	MCI prevalence	Crude OR (95% CI)	Model 1^b^ OR (95% CI)	Model 2^c^ OR (95% CI)	Model 3^d^ OR (95% CI)
None-None (*N* = 249)	57 (22.9)^a^	Ref.	Ref.	Ref.	Ref.
None-Active (*N* = 499)	91 (18.2)	0.75 (0.52–1.09)	0.68 (0.46–1.01)	0.71 (0.48–1.06)	0.72 (0.48–1.08)
Active-None (*N* = 302)	60 (19.9)	0.84 (0.56–1.26)	0.77 (0.50–1.19)	0.75 (0.49–1.17)	0.77 (0.49–1.19)
Active-Active (*N* = 565)	84 (14.9)	0.59 (0.40–0.86)	0.57 (0.38–0.85)	0.60 (0.40–0.90)	0.62 (0.41–0.94)

MCI, mild cognitive impairment. ^a^n (%). ^b^Model 1 was adjusted for age and sex. ^c^Model 2 was adjusted for age, sex, BMI, and years of education. ^d^Model 3 was adjusted for age, sex, BMI, years of education, hypertension, diabetes mellitus, cerebrovascular disease, current and past smoking status, and alcohol intake.

### Comparisons of regional brain volumes and the BDNF, IGF-1, and homocysteine concentrations among groups

Regional brain volumes and the BDNF, IGF-1, and homocysteine concentrations were compared among the four groups (Tables 3, 4). Regarding regional brain volumes (Table 3), the Active-None and Active-Active groups had significantly lower temporal lobe volumes than the None-Active groups (*P* = 0.003). Additionally, the Active-None group had significantly higher hippocampal (Total, Left, or Right) volumes than the None-Active group (*P* = 0.001). However, there were no significant differences in prefrontal and parietal cortical volumes among groups. Moreover, there were no significant differences in BDNF, IGF-1, and homocysteine concentrations among the groups after post-hoc Bonferroni correction (Table 4). Post-hoc power analyses indicated that these sample sizes provided over 95% power to detect the association.

**TABLE 3:** Comparisons of regional brain volumes among groups.

	None-None (*N* = 249)		None-Active (*N* = 497)		Active-None (*N* = 301)		Active-Active (*N* = 561)		*P*-value
	Mean ± SE	95%CI	Mean ± SE	95%CI	Mean ± SE	95%CI	Mean ± SE	95%CI	
Prefrontal, %	4.42 ± 0.02	4.38 – 4.47	4.41 ± 0.02	4.38 – 4.45	4.37 ± 0.02	4.33 – 4.41	4.38 ± 0.02	4.35 – 4.41	0.159
Temporal, %	5.34 ± 0.03	5.28 – 5.40	5.38 ± 0.02	5.34 – 5.42	5.26 ± 0.03 ^b^	5.21 – 5.31	5.26 ± 0.03 ^b^	5.26 – 5.34	0.003
Parietal, %	1.17 ± 0.01	1.16 – 1.19	1.17 ± 0.01	1.15 – 1.18	1.16 ± 0.01	1.14 – 1.17	1.16 ± 0.01	1.15 – 1.17	0.565
**Hippocampus, %**									
Total	0.403 ± 0.002	0.398 – 0.407	0.406 ± 0.002	0.403 – 0.410	0.395 ± 0.002 ^b^	0.390 – 0.399	0.401 ± 0.002	0.398 – 0.405	0.001
Left	0.201 ± 0.001	0.199 – 0.204	0.203 ± 0.001	0.201 – 0.205	0.197 ± 0.001 ^b^	0.195 – 0.200	0.201 ± 0.001	0.199 – 0.203	0.001
Right	0.201 ± 0.001	0.199 – 0.204	0.203 ± 0.001	0.201 – 0.205	0.197 ± 0.001 ^b^	0.195 – 0.199	0.200 ± 0.001	0.199 – 0.202	0.001

Adjusted variables: Age, sex, BMI, years of education, current and past smoking status, hypertension, diabetes mellitus, cerebrovascular disease, and alcohol intake. Multiple comparisons were adjusted using post-hoc Bonferroni correction. ^b^Significant difference compared to the None-Active group.

**TABLE 4:** Comparisons of the BDNF, IGF-1, homocysteine concentrations among groups.

	None-None (*N* = 249)		None-Active (*N* = 499)		Active-None (*N* = 302)		Active-Active (*N* = 565)		*P*-value
Mean ± SE	95%CI	Mean ± SE	95%CI	Mean ± SE	95%CI	Mean ± SE	95%CI		
BDNF, pg/mL	17574 ± 328	16931 – 18218	17893 ± 233	17436 – 18350	17705 ± 298	17119 – 18290	17875 ± 218	17447 – 18303	0.829
IGF-1, ng/mL	90.82 ± 1.83	87.23 – 94.42	95.05 ± 1.30	92.51 – 97.60	90.50 ± 1.66	87.24 – 93.76	95.06 ± 1.21	92.68 – 97.45	0.038
Homocysteine, μmol/L	10.06 ± 0.28	9.51 – 10.61	9.70 ± 0.20	9.30 – 10.08	10.45 ± 0.26	9.94 – 10.95	9.63 ± 0.19	9.26 – 10.00	0.048

BDNF, brain-derived neurotrophic factor; IGF-1, Insulin-like growth factor-1. Adjusted variables: Age, sex, BMI, years of education, current and past smoking status, hypertension, diabetes mellitus, cerebrovascular disease, and alcohol intake. Multiple comparisons were adjusted using post-hoc Bonferroni correction.

## Discussion

The purpose of the present study was to investigate the association between exercise habits in adolescence and old age and the prevalence of MCI in community-dwelling older adults. Our analysis demonstrated that cultivating exercise habits in both adolescence and old age is associated with a lower risk of MCI compared to a lack of exercise habits in both periods. Notably, changes in brain volume and concentrations of key biochemical markers associated with cognitive function are not necessarily correlated with this reduced risk of MCI.

In this study, we used a simple qualitative questionnaire to collect and classify exercise habits and found that having exercise habits in adolescence and old age is associated with a low risk of MCI. However, it should be noted that as the questionnaire was administered to help the participants recall their exercise habits in their adolescence about half a century ago, the possibility of recall bias cannot be ignored. However, as we mentioned before, since Bukatsudo is part of the school education framework, students who participated in these activities engaged in sports regularly and quantitatively. Therefore, we assumed that recall bias in this study was limited. Thus, the simple qualitative questionnaire used in this study is largely valid for assessing exercise habits. Furthermore, it should be noted that it is difficult for doctors to obtain detailed data on exercise history in clinical consultation. Therefore, the results of the simple qualitative questionnaire were considered a reference for indicating the risk of MCI in clinical practice.

Exercise habits in both adolescence and old age may have an additive effect on the maintenance of cognitive function in older adults. The results of this study suggest that the combination of exercise habits in adolescence and old age reduces the risk of MCI. Several previous reviews have shown that engaging in physical activity, a factor related to cognitive reserve (Allegri et al., 2010), in early life (≤ 30 years) could delay cognitive decline in later life (Greene et al., 2019). In addition, growing evidence indicates that having exercise habits in later life can reduce the risk of developing incident MCI in older adults (Krell-Roesch et al., 2016; Ogino et al., 2019). Therefore, increased cognitive reserve and decreased cognitive decline may prevent MCI in older adults. Individuals who exercise in both adolescence and old age may develop lifelong exercise habits (Jose et al., 2011; Sylvester et al., 2020), which may lead to improved cognitive function in older adults (Middleton et al., 2010; Ogino et al., 2019). Jose et al. specifically found that engaging in sports outside of school during adolescence was associated with maintaining physical activity into adulthood (Jose et al., 2011). Therefore, adopting exercise habits in both adolescence and old age may play a role in cognitive function in later life.

While older adults with exercise habits in both adolescence and old age had a lower prevalence of MCI compared to those without exercise habits in either period, there were no significant differences in the volumes of specific brain regions associated with cognitive function, or in the blood levels of BDNF, IGF-1, or homocysteine between the Active-Active group and the None-None group of older adults. These results suggest that exercise may contribute to cognitive preservation through mechanisms independent of structural brain changes or specific biochemical markers. One possible explanation for this finding is that long-term exercise activates neural circuits and improves synaptic plasticity and neural efficiency, which can positively impact cognitive health in ways that are not directly measurable through brain volume or biochemical markers. Gomez et al. provided evidence that habitual exercise enhances synaptic plasticity, further supporting this hypothesis (Gomez-Pinilla and Hillman, 2013). Although regular physical activity is known to increase BDNF level (Vecchio et al., 2018), our study did not find significant associations between exercise habits and BDNF levels, warranting further research into other neurotrophic factors and neurotransmitters to better understand the mechanisms of cognitive aging.

This study has several limitations. As the participants were selected only from urban areas (Bunkyo-Ku, Tokyo, Japan), the possibility of selection bias cannot be ruled out. A previous advanced study showed that the median MoCA-J score and years of education in a local town (Sasaguri district, Fukuoka, Japan) were 22 and 12 years, respectively (Narazaki et al., 2013). However, data from the Bunkyo Health Study, which was used for the present study, indicated that the median MoCA-J score and years of education for Bunkyo City are 26 and 14 years, respectively (Table 1). The type, frequency, or intensity of exercise were not considered in this study. Given that previous studies have indicated that different types (Dunsky et al., 2017; Voelcker-Rehage and Niemann, 2013) and intensities (Coetsee and Terblanche, 2017) of exercise have distinct effects on cognitive function, further research in which different aspects of exercise are evaluated are needed to corroborate the findings of the present study. Another limitation was the relatively small sample size of the None-Active and Active-None groups for the subgroup analyses. It is important to consider the power of the analysis, especially when working with a small number of subjects, which may not reflect the true prevalence. To address this, future studies should employ a longitudinal design and include replication in other cohorts. We used the MoCA-J screening test to detect MCI, without incorporating clinical diagnosis or functional and emotional testing in this study. Another important consideration is that incorporating cognitive symptoms into the assessment is also important. This aspect will be explored further in future research. Moreover, only one neuroradiologist was involved in the MRI assessment. While this approach ensured consistency in image interpretation, the absence of a second reviewer may have introduced a potential for bias. Finally, given that this was a cross-sectional study, the issue of reverse causality should be considered. Therefore, further prospective and interventional studies are needed to clarify the association between exercise habits in adolescence and old age and the prevalence of MCI later in life.

In conclusion, this study demonstrated that having exercise habits both in adolescence (13–18 years) and old age (65–84 years) is associated with a reduced risk of MCI among community-dwelling older adults. However, the results of brain volumes and biochemical markers did not align with the differences in cognitive function, highlighting the complexity of the factors that influence cognitive health in old age. In addition, the simple questionnaire used in this study, which distinguishes whether participants have exercise habits in adolescence or old age or not, may serve as a useful reference tool for assessing the risk of MCI in clinical practice.

## Data Availability

Some or all datasets generated and/or analyzed during the current study are not publicly available as at the time of publication they were undergoing analysis, however, they can be obtained from the corresponding author upon reasonable request.
