# Supplementary material for: Association between exercise habits in adolescence and old age and the risk of mild cognitive impairment: the Bunkyo health study
Source: Front Aging Neurosci. 2024 Nov 11;16:1456665. doi: 10.3389/fnagi.2024.1456665 (PMC11586344; doi:10.3389/fnagi.2024.1456665)
Supplement: Supplementary file 1 [file Table_1.docx]

Supplementary Table S1. The cumulate number of people for each sport in adolescence exercise habits, called "Bukatsudo."

|  | Total (N=1615) | Men (N=677) | Women (N=918) |
| --- | --- | --- | --- |
| No exercise | 737 (45.6) | 250 (36.5) | 487 (52.3) |
| Volleyball | 190 (11.8) | 38 (5.6) | 152 (16.3) |
| Baseball or Softball | 139 (8.6) | 99 (14.5) | 40 (4.3) |
| Tennis | 131 (8.1) | 45 (6.6) | 86 (9.2) |
| Basketball | 120 (7.4) | 65 (9.5) | 55 (5.9) |
| Table tennis | 118 (7.3) | 46 (6.7) | 72 (7.7) |
| Track & Field | 73 (4.5) | 43 (6.3) | 30 (3.2) |
| Judo | 50 (3.1) | 50 (7.3) | 0 |
| Swimming | 46 (2.8) | 30 (4.4) | 16 (1.7) |
| Gymnastics | 43 (2.7) | 11 (1.6) | 32 (3.4) |
| Mountaineering | 37 (2.3) | 21 (3.1) | 16 (1.7) |
| Soccer | 23 (1.4) | 23 (3.4) | 0 |
| Kendo | 19 (1.2) | 16 (2.3) | 3 (0.3) |
| Ski and Skating | 14 (0.9) | 8 (1.2) | 6 (0.6) |
| Dance | 13 (0.8) | 0 | 13 (1.4) |
| Rugby | 12 (0.7) | 12 (1.8) | 0 |
| Badminton | 7 (0.4) | 3 (0.4) | 4 (0.4) |
| Kyudo | 6 (0.4) | 5 (07) | 1 (0.1) |
| Boxing | 6 (0.4) | 6 (0.9) | 0 |
| Rowing and Sailing | 5 (0.3) | 4 (0.6) | 1 (0.1) |
| Ice hockey | 4 (0.2) | 4 (0.6) | 0 |
| Karate | 4 (0.2) | 4 (0.6) | 0 |
| Handball | 4 (0.2) | 4 (0.6) | 0 |
| Wrestling | 4 (0.2) | 4 (0.6) | 0 |
| Golf | 3 (0.2) | 2 (0.3) | 1 (0.1) |
| Equestrian art | 2 (0.1) | 2 (0.3) | 0 |
| American football | 1 (0.1) | 1 (0.1) | 0 |
| Aviation | 1 (0.1) | 1 (0.1) | 0 |
| Weightlifting | 1 (0.1) | 1 (0.1) | 0 |

Supplementary Table S2. The cumulate number of people for each sport in older adults.

|  | Total (N=1615) | Men (N=684) | Women (N=931) |
| --- | --- | --- | --- |
| No Exercise | 631 (39.1) | 292 (42.7) | 339 (36.4) |
| Walking | 289 (17.9) | 166 (24.3) | 123 (13.2) |
| Calisthenics | 189 (11.7) | 37 (5.4) | 152 (16.3) |
| Golf | 112 (6.9) | 87 (12.7) | 25 (2.7) |
| Resistance training | 106 (6.6) | 56 (8.2) | 50 (5.4) |
| Health club activities | 105 (6.5) | 26 (3.8) | 79 (8.5) |
| Stretching | 92 (5.7) | 23 (3.4) | 69 (7.4) |
| Swimming | 77 (4.8) | 29 (4.2) | 48 (5.2) |
| Yoga and Pilates | 63 (3.9) | 6 (0.9) | 57 (6.1) |
| Aqua exercise | 61 (3.8) | 20 (2.9) | 41 (4.4) |
| Tennis | 60 (3.7) | 29 (4.2) | 31 (3.3) |
| Aerobic gymnastics | 55 (3.4) | 8 (1.2) | 47 (5.0) |
| Social dance | 45 (2.8) | 7 (1.0) | 38 (4.1) |
| Running | 41 (2.5) | 31 (4.5) | 10 (1.1) |
| Tai Chi and Qi gong | 32 (2.0) | 1 (0.1) | 31 (3.3) |
| Table tennis | 23 (1.4) | 7 (1.0) | 16 (1.7) |
| Cycling exercise | 18 (1.1) | 8 (1.2) | 10 (1.1) |
| Mountaineering | 15 (0.9) | 10 (1.5) | 5 (0.5) |
| Volleyball | 10 (0.6) | 2 (0.3) | 8 (0.9) |
| Budo | 7 (0.4) | 3 (0.4) | 4 (0.4) |
| Badminton | 5 (0.3) | 3 (0.4) | 2 (0.2) |
| Table top curling | 5 (0.3) | 1 (0.1) | 4 (0.4) |
| Ski | 5 (0.3) | 2 (0.3) | 3 (0.3) |
| Basketball | 4 (0.2) | 4 (0.6) | 0 |
| Baseball | 4 (0.2) | 4 (0.6) | 0 |
| Boxing and Boxercise | 4 (0.2) | 3 (0.4) | 1 (0.1) |
| Ballet | 3 (0.2) | 0 | 3 (0.3) |
| Shooting and Darts | 3 (0.2) | 2 (0.3) | 1 (0.1) |
| Bowling | 3 (0.2) | 2 (0.3) | 1 (0.1) |
| Sailing and Rowing | 3 (0.2) | 3 (0.4) | 0 |
| Horse riding | 1 (0.1) | 1 (0.1) | 0 |
| Ice hockey | 1 (0.1) | 1 (0.1) | 0 |
| Japanese croquet | 1 (0.1) | 0 | 1 (0.1) |
| Dodgeball | 1 (0.1) | 1 (0.1) | 0 |

Supplementary Table S3. Analysis of covariance statistics details for comparisons of prefrontal cortex volume among exercise groups.

|  | Sum of Squares | df | Mean Square | F | P-value | Partial Eta Squared |
| --- | --- | --- | --- | --- | --- | --- |
| Exercise groups | 0.715 | 3 | 0.238 | 1.729 | 0.159 | 0.003 |
| Age | 25.698 | 1 | 25.698 | 186.541 | 0 | 0.105 |
| Sex | 1.807 | 1 | 1.807 | 13.115 | 0 | 0.008 |
| Body mass index | 10.794 | 1 | 10.794 | 78.356 | 0 | 0.047 |
| Education status | 0.056 | 1 | 0.056 | 0.407 | 0.524 | 0 |
| Past smoking status | 0 | 1 | 0 | 0.003 | 0.953 | 0 |
| Current smoking status | 0.047 | 1 | 0.047 | 0.34 | 0.56 | 0 |
| Hypertension | 2.526 | 1 | 2.526 | 18.337 | 0 | 0.011 |
| Diabetes mellitus | 1.221 | 1 | 1.221 | 8.863 | 0.003 | 0.006 |
| Cerebrovascular disease | 0.003 | 1 | 0.003 | 0.022 | 0.883 | 0 |
| Alcohol intake | 1.196 | 1 | 1.196 | 8.684 | 0.003 | 0.005 |
| Error | 219.59 | 1594 | 0.138 |  |  |  |
| Adjusted R Squared = 0.234 | | | | | | |

Supplementary Table S4. Analysis of covariance statistics details for comparisons of parietal cortex volume among exercise groups.

|  | Sum of Squares | df | Mean Square | F | P-value | Partial Eta Squared |
| --- | --- | --- | --- | --- | --- | --- |
| Exercise groups | 0.031 | 3 | 0.01 | 0.679 | 0.565 | 0.001 |
| Age | 0.937 | 1 | 0.937 | 61.303 | 0 | 0.037 |
| Sex | 0.253 | 1 | 0.253 | 16.546 | 0 | 0.01 |
| Body mass index | 0.851 | 1 | 0.851 | 55.701 | 0 | 0.034 |
| Education status | 0.003 | 1 | 0.003 | 0.196 | 0.658 | 0 |
| Past smoking status | 0.001 | 1 | 0.001 | 0.045 | 0.833 | 0 |
| Current smoking status | 0.005 | 1 | 0.005 | 0.334 | 0.564 | 0 |
| Hypertension | 0.195 | 1 | 0.195 | 12.748 | 0 | 0.008 |
| Diabetes mellitus | 0.012 | 1 | 0.012 | 0.78 | 0.377 | 0 |
| Cerebrovascular disease | 0.001 | 1 | 0.001 | 0.041 | 0.84 | 0 |
| Alcohol intake | 0.052 | 1 | 0.052 | 3.38 | 0.066 | 0.002 |
| Error | 24.358 | 1594 | 0.015 |  |  |  |
| Adjusted R Squared = 0.137 | | | | | | |

Supplementary Table S5. Analysis of covariance statistics details for comparisons of temporal lobe volume among exercise groups.

|  | Sum of Squares | df | Mean Square | F | P-value | Partial Eta Squared |
| --- | --- | --- | --- | --- | --- | --- |
| Exercise groups | 3.13 | 3 | 1.043 | 4.637 | 0.003 | 0.009 |
| Age | 49.835 | 1 | 49.835 | 221.492 | 0 | 0.122 |
| Sex | 3.723 | 1 | 3.723 | 16.549 | 0 | 0.01 |
| Body mass index | 9.133 | 1 | 9.133 | 40.593 | 0 | 0.025 |
| Education status | 0.031 | 1 | 0.031 | 0.14 | 0.709 | 0 |
| Past smoking status | 0.02 | 1 | 0.02 | 0.091 | 0.763 | 0 |
| Current smoking status | 0.229 | 1 | 0.229 | 1.016 | 0.314 | 0.001 |
| Hypertension | 2.526 | 1 | 2.526 | 11.226 | 0.001 | 0.007 |
| Diabetes mellitus | 2.627 | 1 | 2.627 | 11.677 | 0.001 | 0.007 |
| Cerebrovascular disease | 0.055 | 1 | 0.055 | 0.245 | 0.621 | 0 |
| Alcohol intake | 1.095 | 1 | 1.095 | 4.869 | 0.027 | 0.003 |
| Error | 358.647 | 1594 | 0.225 |  |  |  |
| Adjusted R Squared = 0.229 | | | | | | |

Supplementary Table S6. Analysis of covariance statistics details for comparisons of total hippocampus volume among exercise groups.

|  | Sum of Squares | df | Mean Square | F | P-value | Partial Eta Squared |
| --- | --- | --- | --- | --- | --- | --- |
| Exercise groups | 0.024 | 3 | 0.008 | 5.562 | 0.001 | 0.01 |
| Age | 0.415 | 1 | 0.415 | 285.904 | 0 | 0.152 |
| Sex | 0.052 | 1 | 0.052 | 36.03 | 0 | 0.022 |
| Body mass index | 0.057 | 1 | 0.057 | 39.37 | 0 | 0.024 |
| Education status | 0 | 1 | 0 | 0.1 | 0.752 | 0 |
| Past smoking status | 0.002 | 1 | 0.002 | 1.659 | 0.198 | 0.001 |
| Current smoking status | 0.002 | 1 | 0.002 | 1.563 | 0.211 | 0.001 |
| Hypertension | 0.006 | 1 | 0.006 | 3.918 | 0.048 | 0.002 |
| Diabetes mellitus | 0.008 | 1 | 0.008 | 5.321 | 0.021 | 0.003 |
| Cerebrovascular disease | 0 | 1 | 0 | 0.216 | 0.643 | 0 |
| Alcohol intake | 0.003 | 1 | 0.003 | 2.093 | 0.148 | 0.001 |
| Error | 2.313 | 1594 | 0.001 |  |  |  |
| Adjusted R Squared = 0.261 | | | | | | |

Supplementary Table S7. Analysis of covariance statistics details for comparisons of right hippocampus volume among exercise groups.

|  | Sum of Squares | df | Mean Square | F | P-value | Partial Eta Squared |
| --- | --- | --- | --- | --- | --- | --- |
| Exercise groups | 0.006 | 3 | 0.002 | 5.256 | 0.001 | 0.01 |
| Age | 0.104 | 1 | 0.104 | 275.519 | 0 | 0.147 |
| Sex | 0.013 | 1 | 0.013 | 33.619 | 0 | 0.021 |
| Body mass index | 0.015 | 1 | 0.015 | 39.709 | 0 | 0.024 |
| Education status | 4.83E-05 | 1 | 4.83E-05 | 0.128 | 0.72 | 0 |
| Past smoking status | 0.001 | 1 | 0.001 | 3.389 | 0.066 | 0.002 |
| Current smoking status | 0 | 1 | 0 | 1.093 | 0.296 | 0.001 |
| Hypertension | 0.002 | 1 | 0.002 | 4.218 | 0.04 | 0.003 |
| Diabetes mellitus | 0.002 | 1 | 0.002 | 4.381 | 0.036 | 0.003 |
| Cerebrovascular disease | 4.76E-05 | 1 | 4.76E-05 | 0.126 | 0.722 | 0 |
| Alcohol intake | 0.001 | 1 | 0.001 | 3.734 | 0.053 | 0.002 |
| Error | 0.6 | 1594 | 0 |  |  |  |
| Adjusted R Squared = 0.256 | | | | | | |

Supplementary Table S8. Analysis of covariance statistics details for comparisons of left hippocampus volume among exercise groups.

|  | Sum of Squares | df | Mean Square | F | P-value | Partial Eta Squared |
| --- | --- | --- | --- | --- | --- | --- |
| Exercise groups | 0.006 | 3 | 0.002 | 5.282 | 0.001 | 0.01 |
| Age | 0.104 | 1 | 0.104 | 265.52 | 0 | 0.143 |
| Sex | 0.014 | 1 | 0.014 | 34.539 | 0 | 0.021 |
| Body mass index | 0.014 | 1 | 0.014 | 34.899 | 0 | 0.021 |
| Education status | 2.57E-05 | 1 | 2.57E-05 | 0.066 | 0.798 | 0 |
| Past smoking status | 0 | 1 | 0 | 0.457 | 0.499 | 0 |
| Current smoking status | 0.001 | 1 | 0.001 | 1.912 | 0.167 | 0.001 |
| Hypertension | 0.001 | 1 | 0.001 | 3.236 | 0.072 | 0.002 |
| Diabetes mellitus | 0.002 | 1 | 0.002 | 5.716 | 0.017 | 0.004 |
| Cerebrovascular disease | 0 | 1 | 0 | 0.298 | 0.585 | 0 |
| Alcohol intake | 0 | 1 | 0 | 0.795 | 0.373 | 0 |
| Error | 0.623 | 1594 | 0 |  |  |  |
| Adjusted R Squared = 0.246 | | | | | | |

Supplementary Table S9. Analysis of covariance statistics details for comparisons of BDNF concentration among exercise groups.

|  | Sum of Squares | df | Mean Square | F | P-value | Partial Eta Squared |
| --- | --- | --- | --- | --- | --- | --- |
| Exercise groups | 22409253.3 | 3 | 7469751.09 | 0.283 | 0.837 | 0.001 |
| Age | 457882558 | 1 | 457882558 | 17.374 | 0 | 0.011 |
| Sex | 2751331.24 | 1 | 2751331.24 | 0.104 | 0.747 | 0 |
| Body mass index | 11325111.8 | 1 | 11325111.8 | 0.43 | 0.512 | 0 |
| Education status | 89123266.2 | 1 | 89123266.2 | 3.382 | 0.066 | 0.002 |
| Past smoking status | 338410911 | 1 | 338410911 | 12.841 | 0 | 0.008 |
| Current smoking status | 1475588.04 | 1 | 1475588.04 | 0.056 | 0.813 | 0 |
| Hypertension | 27142168.9 | 1 | 27142168.9 | 1.03 | 0.31 | 0.001 |
| Diabetes mellitus | 134664958 | 1 | 134664958 | 5.11 | 0.024 | 0.003 |
| Cerebrovascular disease | 2341243.36 | 1 | 2341243.36 | 0.089 | 0.766 | 0 |
| Alcohol intake | 2649755.67 | 1 | 2649755.67 | 0.101 | 0.751 | 0 |
| Error | 4.2192E+10 | 1601 | 26353829.6 |  |  |  |
| Adjusted R Squared = 0.029 | | | | | | |

Supplementary Table S10. Analysis of covariance statistics details for comparisons of IGF-1concentration among exercise groups.

|  | Sum of Squares | df | Mean Square | F | P-value | Partial Eta Squared |
| --- | --- | --- | --- | --- | --- | --- |
| Exercise groups | 6885.604 | 3 | 2295.201 | 2.813 | 0.038 | 0.005 |
| Age | 60444.719 | 1 | 60444.719 | 74.075 | 0 | 0.044 |
| Sex | 12050.639 | 1 | 12050.639 | 14.768 | 0 | 0.009 |
| Body mass index | 14210.677 | 1 | 14210.677 | 17.415 | 0 | 0.011 |
| Education status | 557.077 | 1 | 557.077 | 0.683 | 0.409 | 0 |
| Past smoking status | 1611.287 | 1 | 1611.287 | 1.975 | 0.16 | 0.001 |
| Current smoking status | 437.708 | 1 | 437.708 | 0.536 | 0.464 | 0 |
| Hypertension | 1.367 | 1 | 1.367 | 0.002 | 0.967 | 0 |
| Diabetes mellitus | 1438.786 | 1 | 1438.786 | 1.763 | 0.184 | 0.001 |
| Cerebrovascular disease | 7.345 | 1 | 7.345 | 0.009 | 0.924 | 0 |
| Alcohol intake | 54.444 | 1 | 54.444 | 0.067 | 0.796 | 0 |
| Error | 1304776.45 | 1599 | 815.995 |  |  |  |
| Adjusted R Squared = 0.071 | | | | | | |

Supplementary Table S11. Analysis of covariance statistics details for comparisons of homocysteine concentration among exercise groups.

|  | Sum of Squares | df | Mean Square | F | P-value | Partial Eta Squared |
| --- | --- | --- | --- | --- | --- | --- |
| Exercise groups | 153.398 | 3 | 51.133 | 2.64 | 0.048 | 0.005 |
| Age | 302.691 | 1 | 302.691 | 15.626 | 0 | 0.01 |
| Sex | 1462.269 | 1 | 1462.269 | 75.487 | 0 | 0.045 |
| Body mass index | 0.396 | 1 | 0.396 | 0.02 | 0.886 | 0 |
| Education status | 32.4 | 1 | 32.4 | 1.673 | 0.196 | 0.001 |
| Past smoking status | 210.527 | 1 | 210.527 | 10.868 | 0.001 | 0.007 |
| Current smoking status | 9.636 | 1 | 9.636 | 0.497 | 0.481 | 0 |
| Hypertension | 0.722 | 1 | 0.722 | 0.037 | 0.847 | 0 |
| Diabetes mellitus | 0.024 | 1 | 0.024 | 0.001 | 0.972 | 0 |
| Cerebrovascular disease | 0.221 | 1 | 0.221 | 0.011 | 0.915 | 0 |
| Alcohol intake | 31.752 | 1 | 31.752 | 1.639 | 0.201 | 0.001 |
| Error | 31013.172 | 1601 | 19.371 |  |  |  |
| Adjusted R Squared = 0.098 | | | | | | |
